# Identification of Soybean Mutant Lines Based on Dual-Branch CNN Model Fusion Framework Utilizing Images from Different Organs

**DOI:** 10.3390/plants12122315

**Published:** 2023-06-14

**Authors:** Guangxia Wu, Lin Fei, Limiao Deng, Haoyan Yang, Meng Han, Zhongzhi Han, Longgang Zhao

**Affiliations:** 1 College of Agronomy, Qingdao Agricultural University, Qingdao 266109, China; wuguangxia2020@163.com; 2 Academy of Dongying Efficient Agricultural Technology and Industry on Saline and Alkaline Land in Collaboration with Qingdao Agricultural University, Dongying 257091, China; denglm68@163.com; 3 Qingdao Key Laboratory of Specialty Plant Germplasm Innovation and Utilization in Saline Soils of Coastal Beach, Qingdao Agricultural University, Qingdao 266109, China; 4 College of Grassland Science, Qingdao Agricultural University, Qingdao 266109, China; 5 College of Science and Information, Qingdao Agricultural University, Qingdao 266109, China; lsxbs1997@163.com; 6 Rural Revitalization Service Center, Shizhong District, Zaozhuang 277000, China; hotmeng@163.com

**Keywords:** soybean, mutant lines, identification, dual-convolutional neural network, feature fusion

## Abstract

The accurate identification and classification of soybean mutant lines is essential for developing new plant varieties through mutation breeding. However, most existing studies have focused on the classification of soybean varieties. Distinguishing mutant lines solely by their seeds can be challenging due to their high genetic similarities. Therefore, in this paper, we designed a dual-branch convolutional neural network (CNN) composed of two identical single CNNs to fuse the image features of pods and seeds together to solve the soybean mutant line classification problem. Four single CNNs (AlexNet, GoogLeNet, ResNet18, and ResNet50) were used to extract features, and the output features were fused and input into the classifier for classification. The results demonstrate that dual-branch CNNs outperform single CNNs, with the dual-ResNet50 fusion framework achieving a 90.22 ± 0.19% classification rate. We also identified the most similar mutant lines and genetic relationships between certain soybean lines using a clustering tree and t-distributed stochastic neighbor embedding algorithm. Our study represents one of the primary efforts to combine various organs for the identification of soybean mutant lines. The findings of this investigation provide a new path to select potential lines for soybean mutation breeding and signify a meaningful advancement in the propagation of soybean mutant line recognition technology.

## 1. Introduction

As a crucial grain, oil, and forage crop, soybean (*Glycine max* L. Merr.) holds a significant strategic position in the economic development of China. Rich in oil, protein, and various essential nutrients, soybean plays a prominent role in human and animal diets [1,2]. Currently, the capacity of soybean self-sufficiency is very low in China, with approximately 80–85% of soybeans being imported [3]. This situation has serious implications for China’s food security. Therefore, there is an urgent need to develop new soybean varieties that offer high productivity and excellent quality to address the inadequacies of China’s soybean seed industry.

Cultivar choice and breeding are widely acknowledged as the primary methods for enhancing crop productivity [4]. Mutation breeding is known to easily achieve the development of new variations in a shorter period, which make it widely used as an attractive advancement for soybean breeding programs. However, one drawback of mutation breeding is the potential for creating numerous lines that are highly similar across different classes, making it essential to conduct thorough screening and selection processes to identify and eliminate highly similar lines. Therefore, the accurate identification of soybean cultivars, particularly mutant lines, is essential for evaluating, selecting, and producing new soybean varieties [5]. Furthermore, soybean cultivar recognition also facilitates the study of plant phenotypes [6]. At present, traditional methods of mutant line identification include morphological observation, physiological and biochemical detection, and molecular marker analysis [7,8]. However, these methods have their limitations. Morphological observation requires extensive experience and knowledge, is highly repetitive and labor-intensive, and its accuracy and consistency are not always guaranteed. Biochemical analysis techniques are often destructive and costly, while developing and screening primers for molecular markers can be challenging. By achieving the intelligent recognition of soybean mutant lines, breeders could significantly reduce their workload and obtain more efficient and objective identification results. Therefore, there is a need to explore a rapid, cost-effective, and accurate method to improve the efficiency of soybean mutant line identification. Convolutional neural network (CNN) is a prominent deep learning architecture that can learn features automatically from large and complex databases by processing structured arrays of data with various deep learning architectures [9]. Deep learning has emerged as a prominent technology within the domain of artificial intelligence and has rapidly advanced in recent years. It has been extensively utilized in diverse fields such as product sorting [10], behavior analysis [11,12], food [13,14], medicine [15], and other fields. Deep learning has been proven to have exceptional capabilities in addressing real-life problems. For instance, the automated damage diagnosis of concrete jack arch beams using optimized deep stacked autoencoders and multi-sensor fusion [16] and the torsional capacity evaluation of RC beams using an improved bird-swarm-algorithm-optimized 2D convolutional neural network, which has successfully detected structural damage even under limited sensors and high levels of uncertainties [17].

Over the past decade, image-based recognition methods of agricultural products have achieved significant success with the aid of computer vision and deep learning technologies. For instance, Zhou, et al. [18] proposed a CNN-ATT model to classify wheat kernels into 30 categories, achieving an accuracy of 93.01%. Similarly, Zhang, et al. [19] utilized hyperspectral imaging and deep CNN to classify four corn seed varieties and demonstrated that CNN outperforms KNN and SVM models with a testing accuracy rate of 94.4%. Additionally, Yang, et al. [20] improved the VGG16 model to classify 12 peanut varieties successfully. In the field of soybean classifying research, Zhu, et al. [21] utilized transfer learning to train six pre-trained models, including AlexNet, ResNet18, Xception, InceptionV3, DenseNet201, and NASNetLarge, to classify ten different soybean seed varieties, achieving a classification accuracy of 97.2% with NASNetLarge architecture. Similarly, Zhu, et al. [22] used hyperspectral imaging coupled with CNNs to classify three soybean seed varieties, achieving a classification accuracy of over 90% for each variety. Li, et al. [23] proposed a one-dimensional CNN combined with hyperspectral imaging to classify four types of soybean varieties, achieving the highest classification accuracy of 98.79%. Recently, Huang, et al. [24] designed a full pipeline for soybean seed classification using Mask R-CNN for image segmentation and a Soybean Network (SNet) for classification. The proposed SNet model achieved an accuracy rate of 96.2% for identifying five classes of one soybean variety, outperforming six previous models. However, these studies have focused on classifying soybean varieties, not mutant lines. Mutant lines are a group of soybean plants that share high genetic similarities. Generally, the differences between varieties are more significant than those between mutant offspring, making it more challenging to classify soybean mutants using convolutional neural networks than traditional variety classification. Furthermore, most existing studies have been based on single-branch models, which are unable to analyze the fusion of features across multiple organ dimensions.

Therefore, in this paper, we design a dual-branch framework to fuse the image features (which are obtained by single CNN method) of pods and seeds together to solve this kind of classification problem. There are no such methods at present to combine features from different organs within a dual-branch framework. We employ four single classical CNNs (AlexNet, GoogLeNet, ResNet18, and ResNet50) to extract features from three different layers. The output features of the same layer from pods and seeds are then fused by a concatenation method. The fused features become a feature vector, which is input into the classifier for classification. The contributions of this article are as follows:

(1) Proposing a dual-branch CNN to fuse the image features of soybean pods and seeds together, achieving comprehensive phenotype integration across dimensions for the accurate identification and classification of soybean mutant lines.

(2) Identifying the most similar mutant lines and genetic relationships between certain soybean lines using a clustering tree and t-distributed stochastic neighbor embedding algorithm.

(3) Representing one of the primary efforts to combine various organs for the identification of soybean mutant lines and providing a new path to select potential lines for soybean mutation breeding and a meaningful advancement in the propagation of soybean mutant line recognition technology.

## 2. Materials and Methods

### 2.1. Soybean Samples

**Figure 1:**
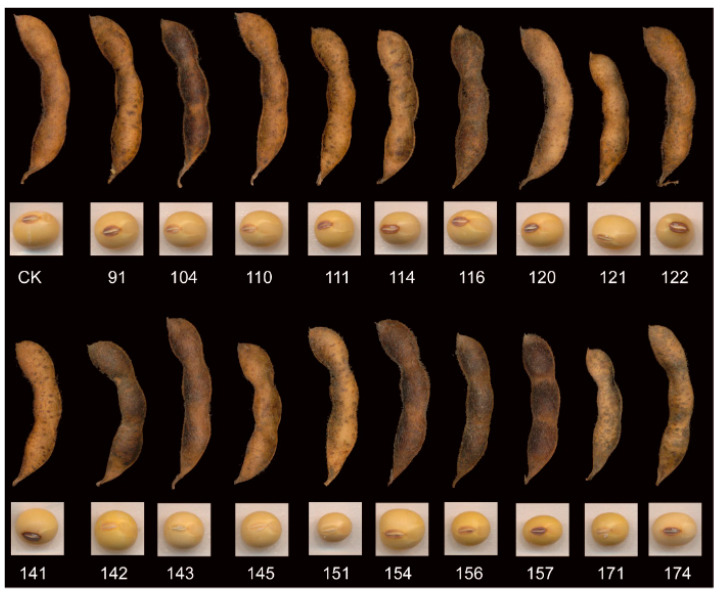
Pod and seed images of 20 types of soybean samples. CK: Hedou12.

The seeds of a Chinese domestic soybean cultivar “Hedou 12” [25] were subjected to radiation using 150, 250, and 350 Gy doses of ^60^Co γ-rays to create a population of mutants. Each group of samples consisted of 500 g soybean seeds, which were subjected to a 30 min irradiation treatment. Nineteen advanced generation mutant lines were then selected from this population. In this study, we examined the untreated “Hedou 12” cultivar, as well as its 19 derivative mutant lines. Figure 1 presents images of pods and seeds for 20 types of soybean samples. The labels and sources of the 20 soybean classes are presented in Table 1.

Taking the number 122 as an example, the notation 14-2-13-2-1 describes the process by which the seeds of Hedou12 were mutated. Specifically, the seeds were exposed to 250 Gy of radiation at the M_0_ generation. From the resulting mutation population, a single plant labeled No.14 was selected at the M_1_ generation, and then No. 14 was planted in rows and a single plant labeled as No.2 was isolated. This process was repeated until the M_5_ generation.

### 2.2. Methods

#### 2.2.1. Image Acquisition

To collect principal images of soybean pods and seeds, a scanner was employed. The soybean samples were placed randomly on the scanner, and the adhered seeds or pods were removed manually. During image acquisition, it was crucial to ensure that the scanner cover plate was fully opened to create a black background. The resulting images of the soybean pods and seeds were transferred to a computer for subsequent processing. The scanner used in this experiment was a Canon Canoscan 8800F, which is a flat CCD scanner with an optical resolution of 4800 dPix9600 dpi, a maximum resolution of 19,200 dpi, and a scanning range of 216 MMX 297 mm. The images were stored on a Lenovo Thinkpad P1 Gen3 computer.

#### 2.2.2. Image Segmentation

**Figure 2:**
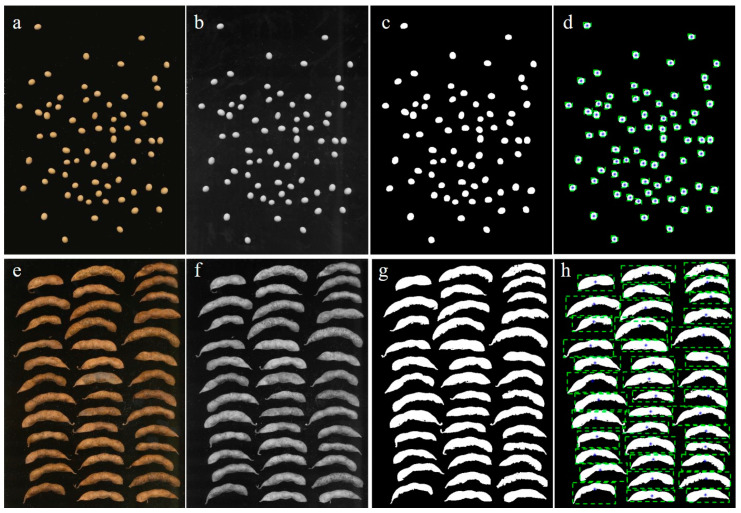
Workflow of soybean seed and pod images segmentation: (**a**,**e**) Original image. (**b**,**f**) Grayscale image. (**c**,**g**) Binarization image. (**d**,**h**) ROI extraction image.

To obtain individual images of soybean seeds and pods without removing the background, an image segmentation step was performed. The process of image segmentation is illustrated in detail in Figure 2. Initially, a series of original principal images were acquired using the scanner (Figure 2a,e). Next, these principal images were converted into grayscale images through grayscale processing (Figure 2b,f). The grayscale images were then used to create binary images, with soybean seed and pod regions represented by “1” and background regions represented by “0”, effectively isolating the seeds and pods from the background (Figure 2c,g). Subsequently, the contour of the connected region was retrieved to obtain the area of the region. A contour box was then selected for a single soybean seed or pod to obtain Figure 2d,h. Finally, the selected soybean seed and pod images within the box were mapped back to the original image and extracted as a single image, which was then saved.

However, image segmentation usually results in individual image pixels being unable to meet the input dimension requirements of convolutional neural networks. Directly enlarging the image size may lead to the loss of genuine size information for individual seeds and pods in the image. To address this issue, we developed a strategy to process the background of individual images. Specifically, we initiated the process by creating a 300 × 300 black background and subsequently assigned a value of 0 to the pixels corresponding to the segmented single image area, in accordance with the black area of the binary image. Finally, we overlaid the processed image onto the 300 × 300 black background image to obtain an “optimized” version of the image. Following the completion of image segmentation processing, this approach yielded 4179 single pod images and 11,247 single seed images. A comprehensive breakdown of the original dataset for every soybean pod and seed type can be found in Table 2.

#### 2.2.3. Image Augmentation

Data augmentation is a vital method for regularization in enhancing the generalization abilities of CNNs when it comes to image classification tasks [26,27]. This method involves the creation of a more extensive and diverse set of training data by randomly transforming images. Due to its high efficacy, data augmentation has become a frequently employed technique for enhancing classification accuracy across a range of image classification tasks [28]. The current study observed an imbalance in the number of pods and seeds among 20 types of soybean materials, as shown in Table 2. It is important to recognize that inadequate data can result in insufficient training of the neural network, as indicated in previous studies [29,30]. Moreover, imbalanced data can pose a potential threat to the classification performance of the neural network, as found in recent research [31,32]. Therefore, to overcome these limitations, we employed data augmentation techniques to rectify the small-scale dataset and class imbalance. Specifically, rotation, shift, flip, and mirror operations were applied to augment the dataset. After augmentation, each class of pods and seeds comprised 1000 individual images, randomized to a proportion of 8:1:1 for the training set, validation set, and test set, respectively.

#### 2.2.4. Dual-Convolution Neural Network Model Fusion Frameworks

**Figure 3:**
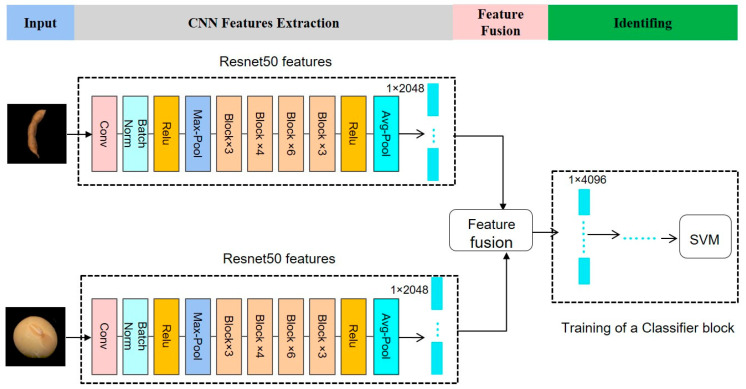
Dual-convolution neural network model fusion framework. Taking the ResNet50-based dual-CNN model fusion strategy as an example.

In this section, we propose four fusion frameworks, namely dual-AlexNet, dual-GoogLeNet, dual-ResNet18, and dual-ResNet50, which were designed to integrate deep features of seed and pod images through dual-CNN models. The current mainstream pre-trained models for transfer learning, including AlexNet, GoogLeNet, and ResNet, had their network parameters pre-trained on the ImageNet dataset, and then were fine-tuned on the dataset used in this study. The fine-tuning process involved freezing the network parameters of several preceding convolution layers and creating a new fully connected layer to be retrained. Feature fusion is an algorithm used to merge independent features into a unique feature to enable easy processing [33]. The ResNet50-based dual-CNN framework is specifically introduced and depicted in Figure 3. The architecture of this framework consists of two identical single ResNet50 models that independently process seed and pod images as input. The image input dimension of each channel was 224 × 224 × 3, and the feature map was extracted from the pre-trained ResNet50 model. The features of both pods and seeds were separately extracted from the Avg_pool layer of a single ResNet50 network, resulting in a 1 × 2048 feature matrix for each. These two feature matrices were then concatenated directly together using a concatenate features method to form a new 1 × 4096 feature matrix, which served as the direct input for the support vector machine (SVM) classifier. The SVM is a strong and effective machine learning model that finds broad applicability across a variety of classification problems [34]. Recognition of soybean mutant lines was achieved through SVM classification. It is noteworthy that the feature extraction process of each dual-CNN comprises the selection of three distinct layers, but only the Avg_pool layer of dual-ResNet50 is depicted in Figure 3 for illustrative purposes. In order to ensure fairness in model comparison, this strategy was adopted by all other model fusion methods. The detailed feature vectors extracted by four single CNN models at three different layers and their fused feature vectors via concatenation are listed in Table 3.

#### 2.2.5. Workflow Diagram

**Figure 4:**
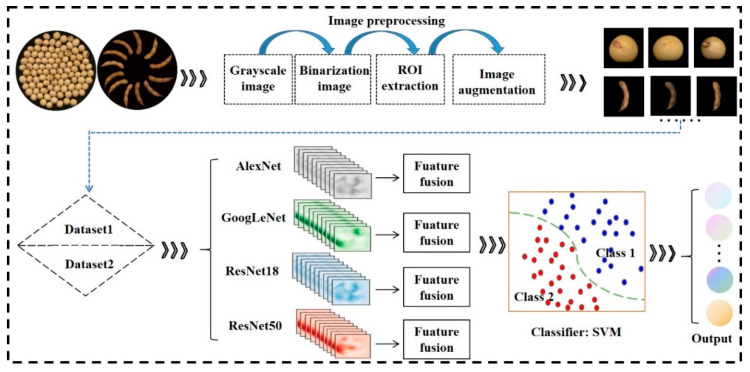
The whole process of soybean mutant lines identification.

Figure 4 depicts a workflow schematic that outlines the methodology employed in this research. The study involved a four-step process, beginning with data collection, which included sample preparation and image dataset collection. Next, only seed or pod images were used as samples to identify soybean mutant lines (not shown in the figure). In this experiment, four classical recognition models, namely AlexNet [35], GoogLeNet [36], ResNet18, and ResNet50 [37], were employed for training and soybean mutant lines classification. Thirdly, four dual-CNN working strategies were implemented. The four pre-trained CNN models from the previous step were directly applied to our proposed dual-CNN fusion models. Each dual-CNN structure involved two identical parallel branches and independently exploited pod and seed datasets for feature extraction. Three different layers of each single CNN were selected for feature extraction. Finally, the extracted features of seed and pod at the same layer, in the same single CNN, were fused and input into the SVM classifier block for soybean line classification. The optimal strategy was selected by analyzing the training results. Four single-CNN models (AlexNet, GoogLeNet, ResNet18, and ResNet50) and the corresponding dual-branch CNN networks were run three times, respectively. All single model sizes and training parameters are shown in Table 4.

## 3. Results and Analysis

### 3.1. Comparison of Different Single Model Training Results

**Figure 5:**
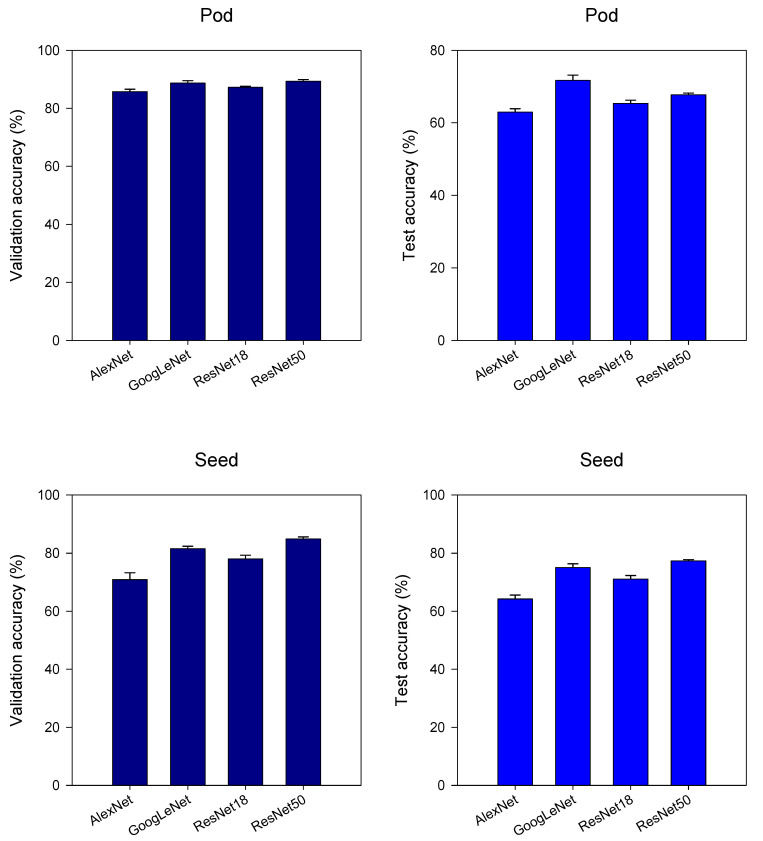
Comparison of accuracy of all single CNN models. The accuracy of the models was the average accuracy of the three instances of model training. Error bars refer to the standard error (SE).

Initially, we solely employed images of soybean seeds or pods as cues to identify mutant lines. All the four single CNN models completed their training after 100 epochs. Figure 5 presents the average validation accuracy and average test accuracy of all the single CNNs. In terms of soybean pods, all four models yielded an average validation accuracy score ranging from 85% to 90%, with ResNet50 boasting the highest accuracy rate of 89.3%. However, when it comes to soybean seeds, the average validation accuracy scores of the same models were comparatively lower, ranging from 70% to 85%. Similar to soybean pods, ResNet50 had the highest validation accuracy performance with a score of 84.8%. During the testing phase, all four models resulted in lower accuracy scores below 80% for both pods and seeds. Specifically, ResNet50 achieved the highest test accuracy score of 77.37% for seeds, while ResNet18 garnered the highest test accuracy score of 71.68% for pods.

### 3.2. Dual Network Selection and Evaluation

Table 5 illustrates the validation and test accuracy of various fusion frameworks. The findings demonstrate that among the selected three different layers for feature extraction and fusion, the four dual-CNNs exhibit improved accuracy compared to their corresponding single CNNs after feature fusion. Specifically, the dual-AlexNet model demonstrated superior classification recognition performance after extracting features of pods and seeds from the fc8 and relu7 layers followed by feature fusion, achieving a validation accuracy exceeding 94% and a testing accuracy exceeding 82%. The dual-GoogLeNet model exhibited strong classification recognition when extracting features of pods and seeds from the inception_5b-output and pool5-7x7_s1 layers and subsequently performing feature fusion, resulting in a validation accuracy surpassing 95% and a testing accuracy exceeding 86%. The dual-ResNet18 model demonstrated superior classification recognition performance after conducting feature extraction on pods and seeds at both the res5b_relu and pool5 layers followed by feature fusion, exhibiting a validation accuracy above 95% and a testing accuracy above 85%. The dual-ResNet50 model showed excellent performance in classification recognition by extracting features of pods and seeds at all three feature extraction layers for performing feature fusion, and achieved a validation accuracy over 90% and a testing accuracy over 80%, respectively. The experimental results demonstrate that the proposed dual-ResNet50 model fusion framework, which fused the features of pod and seed at the average pool layer, attained a classification accuracy higher than the other three proposed dual-CNNs. Specifically, the dual-ResNet50 model achieved the highest test accuracy of 90.22%, which was 2.2% higher than dual-AlexNet, 2.2% higher than dual-GoogLeNet, and 2.47% higher than dual-ResNet18. The dual-ResNet50 proves the effectiveness of the proposed strategy.

**Figure 6:**
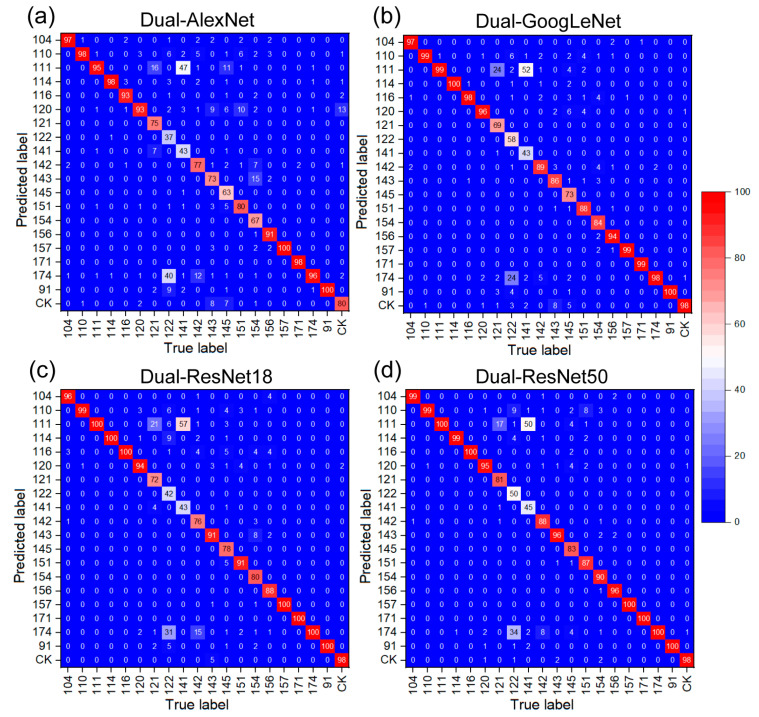
Confusion matrix under different fusion frameworks.

Due to the fact that accuracy is typically used for evaluating models at a global level, it is important to apply the confusion matrix in order to analyze the specific effects of model performance on individual category classification. Each column of the confusion matrix represents the true attribution category, with the total amount of data in each column representing the number of samples in that category (with a total of 100 images per category). Each row represents the predicted category, with the total amount of data in each row showing the predicted number of samples in that category. Diagonal values indicate the number of samples correctly classified, while off-diagonal values indicate the number of samples improperly classified for other categories. Figure 6 shows the confusion matrices for each dual-CNN model, which were built based on the test accuracy of the best feature fusion layer. As shown in Figure 6a, only two samples (91 and 157) were predicted 100% correctly, while sample 122 had the lowest prediction percentage of 37%. Notably, 40% of the images in sample 122 were incorrectly classified as sample 174. Sample 141 also performed poorly with a prediction accuracy of 43%. The remaining mutant lines had prediction accuracies between 63% and 98%. The confusion matrix for the dual-GoogLeNet model in Figure 6b revealed that two samples (91 and 114) were classified 100% correctly, while sample 141 had the poorest prediction accuracy of 43%. The majority of sample 141 (52%) images were misclassified as sample 111. Sample 122 also had a poor prediction accuracy of 58%, with 24% of the images misclassified as sample 174. Moving to the dual-ResNet18 confusion matrix in Figure 6c, the prediction accuracy rates for samples 122 and 141 were below 45%, and 31% of sample 122 and 57% of sample 141 were misclassified as samples 111 and 174, respectively. However, samples 91, 111, 114, 116, 157, 171, and 174 were predicted 100% correctly. In the confusion matrix for the dual-ResNet50 model in Figure 6d, all the classes had greater than 80% prediction accuracy, except for samples 122 (50%) and 141 (45%). Notably, 34% of the sample 122 images were misclassified as sample 174, while 50% of the sample 141 images were misclassified as sample 111. In summary, a significant number of samples 122 and 141 across the four confusion matrices were commonly misclassified as samples 174 and 111, respectively. Moreover, sample 91 was always predicted 100% correctly, suggesting that the non-mutant sample 91 was notably different from the mutant offspring.

In order to provide a comprehensive evaluation of the performance of the four dual-CNN models, we utilized Precision, Recall, and F1-Score as indicators to quantify their classification performances. The evaluation involved four basic parameters, namely true positive (*TP*), true negative (*TN*), false positive (*FP*), and false negative (*FN*). The *TP* parameter represents the number of positive samples correctly identified as positive, while the *TN* parameter represents the number of negative samples correctly identified as negative. On the other hand, the *FP* parameter represents the number of negative samples wrongly identified as positive, and the *FN* parameter represents the number of positive samples wrongly identified as negative. Based on these parameters, the Precision (*P*), Recall (*R*), and *F1*-*Score* are calculated as follows:

(1) $$P=\frac{TP}{TP+FP}$$

(2) $$R=\frac{TP}{TP+FN}$$

(3) $$F1-Score=\frac{2PR}{P+R}$$

Table 6 illustrates the Precision, Recall, and F-score measures of the four distinct fusion frameworks employed in the classification of 20 types of soybeans. The average Precision, Recall, and F-score signify the corresponding mean value of each metric across all categories. From Table 6, it is evident that the dual-ResNet50 architecture outperformed the other three fusion frameworks in terms of accuracy. The average Precision, average Recall, and average F1-score for this framework were 0.9030, 0.9299, and 0.9008, respectively. In summary, the above results show that the proposed dual-ResNet50 fusion framework is superior to the other three dual-CNN frameworks, making it the prime dual-CNN model for classifying soybean mutant lines.

### 3.3. Feature Visualization Analysis

**Figure 7:**
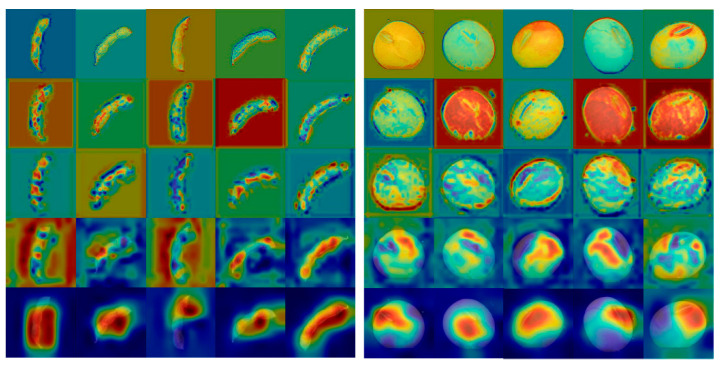
Visualization of Grad-CAM features.

To determine the optimal feature extraction layer, the gradient weighted classes activation mapping (Grad-CAM) [38] method was applied to visualize the seeds and pods on the ResNet50 network. Grad-CAM is a feature visualization technique used to generate a class activation heat map by computing the classification gradients of the convolutional feature maps to identify the most classification-dependent feature locations. The strength of the activation region represents the most critical impact on the classification results. Given that ResNet50 achieves the highest accuracy in the single model test, we selected its conv1, res2c_branch2c, res3c_branch2c, res4c_branch2c, and res5c_branch2c layers for feature extraction. The resulting Grad-CAM visualizations of example images of 5 classes of soybean seed and pod among the 20 classes of samples are presented in Figure 7. The visualization showed that each type of soybean sample exhibited similar patterns in feature visualization. In the shallow layers (conv1, res2c_branch2c, and res3c_branch2c), ResNet50 extracts visual features including contour, color, and edge. As the model layers deepen, the visual features become vague while the abstract information increases. At the res5c_branch2c layer, the activation regions were notably strong, suggesting that with the deepening of layers, the features learned by deep learning become increasingly more representative.

Each column represents a different sample (from left to right: 104, 114, 154, 174, CK) and each row represents a different feature extraction layer (from top to bottom: conv1, res2c_branch2c, res3c_branch2c, res4c_branch2c, and res5c_branch2c).

### 3.4. Clustering Results among Soybean Mutant Lines

**Figure 8:**
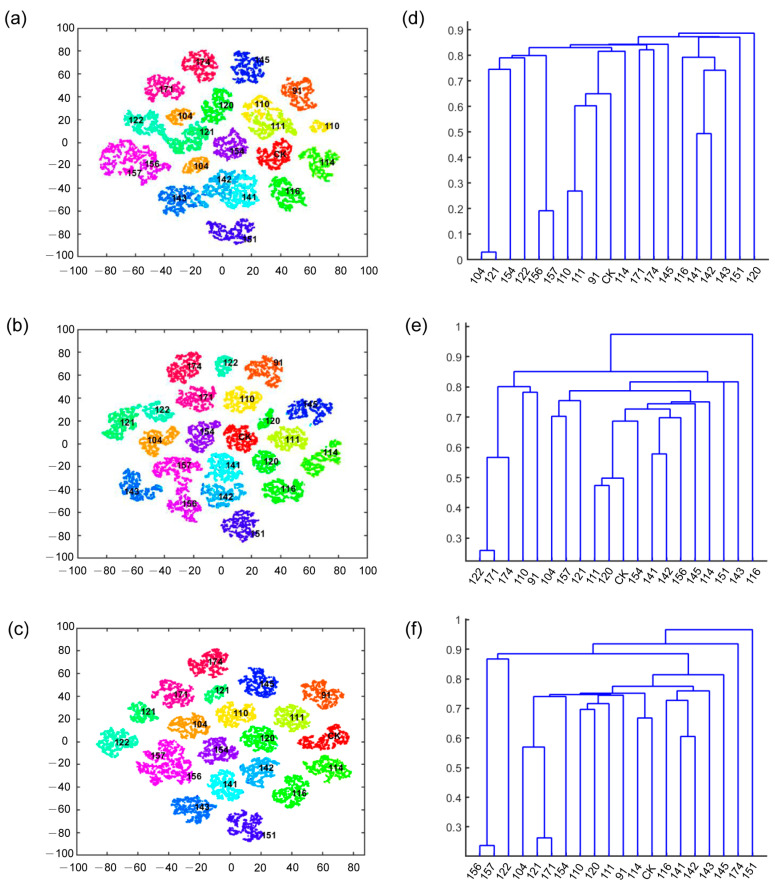
Depth feature clustering results. The 2-D feature visualization using the extracted feature of seeds (**a**) or pods (**b**) or the fused features (**c**). The clustering tree built with the 3-D data of seeds (**d**) or pods (**e**) or both fused (**f**).

To further highlight the discriminative feature learning capacity of the proposed dual-ResNet50 network, we present a two-dimensional feature visualization of 20 classes of soybean samples. The visualization of the feature distribution difference was accomplished by means of the t-distributed stochastic neighbor embedding (t-SNE) algorithm, which is a nonlinear dimensionality reduction tool, well suited for high-dimensional feature visualization through mapping to 2-D or 3-D spaces. As displayed in Figure 8a–c, we obtained the 2-D data visualization following dimensionality reduction. The results reveal that certain samples, such as 156 and 157, as well as 141 and 142, share overlapping clusters. A similar pattern was observed for 110 and 111 samples (Figure 8a), indicating a high level of similarity in the seed features of these overlapping samples. Additionally, it was observed that sample 110 and 104 each exhibited two piles (Figure 8a), suggesting that these samples may not yet be homozygous. Similarly, two piles were also detected in pods 110 and 111 (Figure 8b), suggesting that there might be character segregation in the pods. It is clear that the 2-D feature visualization generated using pods data implies less overlap in the 2-D spatial distributions among the 20 categories than that produced using seeds data (Figure 8a,b). In Figure 8c, similar phenomena were identified using the fusion data of seed and pod. The resultant visualization reveals that the piles of 156 and 157 samples overlap, and two piles are present in 121 samples.

Cluster analysis is employed to classify samples using 3-D data and is often interpreted with the K-means algorithm. Samples with similar features are placed in the same branch of the dendrogram, and differences between groups are defined using heterogeneity or relative distance values. The results of the clustering analysis indicate that the soybean samples can be separated into four categories based on seed data: 143 and 104 samples are classified separately, while 91 and 114 samples constitute the third category, and the remaining samples are grouped into the fourth major category (Figure 8d). Additionally, soybean samples can be roughly divided into three categories based on pod data: 141 samples form a distinct class, and the second category includes samples 111, 116, 142, 151, 114, and 143, with the rest of the samples constituting the third category (Figure 8e). Furthermore, combining seed and pod data reveals that soybean samples can be separated into four categories: samples 122, 151, and 141 exist in separate branches, while the remaining samples are grouped into the fourth major category (Figure 8f). Despite differences in the data used for clustering, samples 156 and 157 always appear together, indicating a close genetic relationship between these soybean lines. These results align with the two-dimensional feature visualization and provide further insight into why these samples are prone to confusion.

## 4. Discussion

### 4.1. Superiority of Dual-Branch CNN over Single Classical CNN

The dual-branch CNN model is a commonly used deep learning model for processing multimodal data, which refers to different types of data generated by multiple sources [39]. In this model, each branch represents an independent CNN model that processes a specific data source. The outputs of these branches are fused and used for classification or regression tasks. Our proposed dual-branch CNN model has two separate branches, each handling a different type of image input. The features extracted from each branch are then combined at a later layer for the final classification. This approach has been shown to outperform the traditional single classical CNN model, which only uses one type of image input. For example, a study by Liu, et al. [40] showed that a dual neural network outperformed the single neural network for the task of recognizing aluminum profile surface defects.

The advantage of the dual-branch CNN model described in this paper is that it can fully utilize the image information of soybean pods and seeds, thereby improving classification performance. At the same time, using multiple classic single CNN models to extract features from different layers and fusing them together can better capture features of different scales and complexities, thereby improving classifier performance. In our study, we specifically mention the dual-ResNet50 framework, which achieves an exciting classification rate of 90.22%. This result is 22.47% higher than the single ResNet50 model used for pod image identification and 12.85% higher than the single ResNet50 model used for seed image identification. This finding has demonstrated the effectiveness of dual-branch CNN models for soybean mutant image classification tasks. However, it is important to note that our model has certain limitations, and future work is needed to optimize it by incorporating weight values to adjust the linear relationships of the fitted data.

### 4.2. Utilization of Clustering Tree

The article reports that a clustering tree was constructed based on the K-means clustering method, which can be utilized for screening promising lines for successful mutation breeding. Clustering analysis is a powerful technique for grouping data points based on their similarities or dissimilarities, and the K-means algorithm is one of the most commonly used clustering methods [41]. By constructing a clustering tree, researchers can visualize the hierarchical structure of the data and identify clusters at different levels of granularity. This can help in identifying potential candidates for mutation breeding, based on their similarity to existing successful lines.

Mutation breeding is an important tool for crop improvement, and has been used to develop new crop varieties with improved traits such as disease resistance, yield, and quality [42]. However, the success rate of mutation breeding is relatively low, due to the low probability of obtaining desirable mutations and the high number of non-target mutations. Therefore, there is a need for efficient screening methods to identify promising lines for further study. Previous research reported constructing a pedigree clustering tree of 20 peanut varieties using the K-means clustering method, which may aid in conducting thorough investigations into the genetic relationships among diverse varieties [43]. In conclusion, the construction of a clustering tree based on the K-means clustering method can be a useful tool for screening promising lines for successful mutation breeding. However, further studies are needed to evaluate the effectiveness of this approach in different crop species and breeding programs.

### 4.3. Significance of Joint Identification of Soybean Mutant Lines

Mutation breeding is a crucial technique in soybean improvement programs to develop new varieties with improved traits, such as yield, disease resistance, and nutrient content [42]. However, the success of mutation breeding largely depends on the accurate identification and classification of mutant lines. Any misclassification or misidentification of mutant lines could lead to the rejection of promising lines and the selection of less desirable ones, which can adversely affect the breeding progress. The use of advanced technology such as deep-learning-based CNN models can help breeders to classify soybean mutant lines rapidly and efficiently. In this study, we propose a dual-branch CNN model that fuses deep learning features from images of soybean pods and seeds. The proposed dual-branch CNN method is among the first attempts to jointly use images from different organs for identifying soybean mutant lines. The identification of confusing mutant lines, which are characterized as difficult to discern or having lower recognition rates, can be vital in screening and subsequent research. This information holds great significance for breeders, allowing them to consistently perform subtraction, and subsequently reduce workload, thereby accelerating the process of breeding screening.

Our study can promote soybean mutant line recognition technology and provide a new path to select elite lines for soybean mutation breeding. Its significance is the joint use of images from different organs for identifying soybean mutant lines, which is a novel approach in the field of soybean mutation breeding. This method can improve the accuracy and efficiency of identifying elite soybean mutant lines, and ultimately contribute to the development of soybean breeding. The use of multiple organs for identification is a more comprehensive approach than relying on a single organ, as different organs may exhibit varying phenotypic traits. The proposed method provides a new perspective for the identification of soybean mutant lines, and it is expected to advance the development of soybean breeding.

## 5. Conclusions

We propose a dual-branch convolutional neural network (CNN) that combines the deep learning features of pod and seed images for the identification and classification of soybean mutant lines. The results show that the proposed dual-branch CNNs outperform the corresponding single classical CNNs, and the dual-ResNet50 fusion framework achieved an exciting classification rate of 90.22%. The clustering tree based on the K-means clustering method can be utilized to screen promising lines for mutation breeding. The significance of jointly using images from different organs for identifying soybean mutant lines is highlighted, and the study sheds light on a promising new direction for the identification of soybean mutant lines. In our future work, we will attempt to classify soybean mutant lines solely based on seeds by taking multiple angle photos of the seed hilum surface using various cameras, such as regular RGB cameras and depth cameras, and by various feature fusion methods.

## Figures and Tables

**Table 1:** Experimental materials for soybean lines identification.

Category	Irradiation Intensity (Gy)	Pedigree Source	Category	Irradiation Intensity (Gy)	Pedigree Source
CK	\	Hedou12	141	250	14-9-2
91	150	1-1-2-1	142	250	3-1-6
104	150	5-1-1-7	143	250	14-2-2
110	150	8-7-2-2	145	150	3-1
111	150	10-3-1	151	250	14-1-11
114	250	3-1-2	154	250	14-1-14
116	250	3-6-2	156	250	14-3-1
120	250	11-2-1-2	157	250	14-8-1
121	250	14-2-1	171	250	14-11
122	250	14-2-13-2-1	174	350	15-3-9

**Table 2:** Number of original pod and seed images collected in the experiment.

Category	Pod	Seed	Category	Pod	Seed
CK	155	377	141	220	752
91	148	820	142	199	674
104	355	375	143	193	694
110	138	620	145	225	427
111	156	518	151	170	652
114	241	577	154	225	444
116	264	411	156	209	663
120	145	565	157	263	479
121	222	457	171	269	488
122	228	436	174	154	818

**Table 3:** Feature vectors extracted by four single CNN models at three different layers and their fused feature vectors via concatenation.

Model	Feature Extraction Layer	Extracted Feature Vector (Pod)	Extracted Feature Vector (Seed)	Fused Feature Vector
Dual-AlexNet	fc8	1 × 4096	1 × 4096	1 × 8192
	relu7	1 × 4096	1 × 4096	1 × 8192
	prob	1 × 20	1 × 20	1 × 40
Dual-GoogLeNet	inception_5b-output	1 × 50,176	1 × 50,176	1 × 100,352
	pool5-7x7_s1	1 × 1024	1 × 1024	1 × 2048
	prob	1 × 20	1 × 20	1 × 40
Dual-ResNet18	res5b_relu	1 × 25,088	1 × 25,088	1 × 50,176
	pool5	1 × 512	1 × 512	1 × 1024
	prob	1 × 20	1 × 20	1 × 40
Dual-ResNet50	activation_48_relu	1 × 50,176	1 × 50,176	1 × 100,352
	avg_pool	1 × 2048	1 × 2048	1 × 4096
	fc1000_softmax	1 × 20	1 × 20	1 × 40

The feature vector extracted from the pod is denoted as 1 × m, and the feature vector extracted from the seed is denoted as 1 × n. Concatenation of the pod and seed feature vectors yields a 1 × (m + n) vector. Taking the ResNet50-based dual-CNN model fusion strategy as an example.

**Table 4:** Parameter values for training convolutional neural network models.

Model	Depth Layer	Size/MB	Batch Size	Learning Rate	Validation Frequency	Input Size
AlexNet	25	227	32	0.0003	64	227 × 227 × 3
GoogLeNet	144	27	32	0.0003	64	224 × 224 × 3
ResNet18	71	44	32	0.0003	64	224 × 224 × 3
ResNet50	177	96	32	0.0003	64	224 × 224 × 3

**Table 5:** Comparison of accuracy of different feature fusion layer combinations and different fusion frameworks.

Accuracy (%)	Replicates	Dual-AlexNet			Dual-GoogLeNet			Dual-ResNet18			Dual-ResNet50		
		fc8	relu7	prob	inception_5b-output	pool5-7x7_s1	prob	res5b_relu	pool5	prob	activation_48_relu	avg_pool	fc1000_softmax
Validation	1	94.10	94.20	88.50	95.95	96.75	91.75	95.80	95.60	90.55	97.50	97.90	93.75
	2	94.10	94.85	87.25	94.85	95.50	91.30	96.15	96.70	90.85	96.20	97.10	93.65
	3	94.85	94.80	88.85	95.80	96.50	91.85	94.25	95.75	88.80	96.70	97.80	93.15
	Average	94.35 ± 0.35	94.62 ± 0.30	88.20 ± 0.69	95.53 ± 0.49	96.25 ± 0.54	91.63 ± 0.24	95.40 ± 0.83	96.02 ± 0.49	90.07 ± 0.90	96.80 ± 0.54	**97.60 ± 0.36**	93.52 ± 0.26
Test	1	82.70	81.75	66.10	86.80	88.35	78.10	85.70	87.40	74.05	89.90	90.30	81.05
	2	83.00	83.25	67.10	85.25	87.00	75.60	87.45	88.40	78.95	87.90	89.95	80.50
	3	83.55	83.25	68.40	87.10	88.70	77.45	84.75	87.45	75.05	88.70	90.40	81.45
	Average	83.08 ± 0.35	82.75 ± 0.71	67.20 ± 0.94	86.38 ± 0.81	88.02 ± 0.73	77.05 ± 1.06	85.97 ± 1.12	87.75 ± 0.46	76.02 ± 2.11	88.83 ± 0.82	**90.22 ± 0.19**	81.00 ± 0.39

The accuracy is represented by the mean plus or minus the standard error. Data in bold indicate optimal.

**Table 6:** Average statistical parameters of four fusion frameworks.

Method	Precision (%)	Recall (%)	F1-Score (%)
Dual-GoogLeNet	88.35	91.18	88.12
Dual-AlexNet	82.7	85.98	82.18
Dual-ResNet18	87.4	90.55	87.01
Dual-ResNet50 (proposed)	90.3	92.99	90.08

## Data Availability

Data are available on request.
